# Water extract of tendril of *Cucurbita Moschata* Duch. suppresses RANKL-induced osteoclastogenesis by down-regulating p38 and ERK signaling

**DOI:** 10.7150/ijms.39622

**Published:** 2020-02-24

**Authors:** Joo-Hee Choi, Ah-Ra Jang, Ha-Na Jeong, Kiok Kim, Young-Min Kim, Jeong-Yong Cho, Jong-Hwan Park

**Affiliations:** 1 Laboratory Animal Medicine, College of Veterinary Medicine and BK 21 PLUS Project Team, Chonnam National University, Gwangju, Republic of Korea.; 2 Laboratory Animal Center, Daegu-Gyeongbuk Medical Innovation Foundation, Daegu, Republic of Korea.; 3 Department of Food Science & Technology, Chonnam National University, Gwangju, Republic of Korea.

**Keywords:** Osteoclast differentiation, RANKL, Tendril of *Cucurbita Moschata* Duch. (TCMD)

## Abstract

**Background:** Pumpkin (*Curcubita sp.*) is a natural product that is commonly used in folk medicine. However, the inhibitory effect and molecular mechanisms of tendril of *Cucurbita Moschata* Duch. (TCMD) on osteoclast differentiation have yet to be clearly elucidated. Thus, the present study aimed to investigate the effect and underlying mechanism of water extract of TCMD on osteoclast differentiation.

**Methods:** Bone marrow-derived macrophages (BMDMs), osteoclast precursors, were cultured with macrophage colony stimulating factor (M-CSF) 30 ng/ml and receptor activator of nuclear factor-kappa B ligand (RANKL) 100 ng/ml for four days. We investigated the effect of TCMD on RANKL-induced osteoclast differentiation, tartrate-resistant acid phosphatase (TRAP) staining, F-actin ring formation, and bone resorption assay. RANKL signaling pathways were determined through Western blotting, and osteoclast differentiation marker genes were confirmed by Real-time PCR.

**Results:** TCMD inhibited the RANKL-induced osteoclast differentiation in a dose-dependent manner without cytotoxicity. Further, F-actin ring formation and bone resorption were reduced by TCMD in RANKL-treated BMDMs. In addition, TCMD decreased the phosphorylation of p38 and ERK as well as the expression of osteoclast-related genes in BMDMs treated with RANKL.

**Conclusion:** These findings suggest that TCMD may have preventive and therapeutic effects for destructive bone diseases.

## Introduction

Bone is a dynamic organ that maintains its structure and function by replacing the old and damaged bone with new bone via bone remodeling. The bone remodeling cycle involves a balanced interaction between osteoclastic bone resorption and osteoblastic bone formation 1. Osteoclasts, multinucleated bone-resorbing cells, are derived from the hematopoietic precursor cells of monocyte/macrophage lineage at various stages, including proliferation, migration, fusion, and activation 2. Osteoclastogenesis is an osteoblast-dependent process that is regulated by two critical cytokines: the macrophage colony-stimulating factor (M-CSF) and receptor activator of nuclear factor-kappa B (NF-κB) ligand (RANKL) 3. M-CSF plays an important role in the survival and proliferation of precursors of the monocyte/macrophage lineage. RANKL is secreted by osteoblasts and binds to its receptor RANK to differentiate osteoclast precursors into osteoclasts 4,5. The binding of RANKL to RANK activates the mitogen-activated protein kinase (MAPK) pathway and NF-κB, and the downstream factors including the nuclear factor of activated T cells c1 (NFATc1), which is the master regulator of osteoclastogenesis 6,7. The activation of these signal molecules results in the differentiation and activation of osteoclasts, which play a role in bone resorption and actin ring formation 8. Abnormal osteoclastogenesis or osteoclast activation leads to an imbalance in bone remodeling, which predominantly causes osteoporosis, rheumatoid arthritis, and periodontitis. In attempts to develop therapeutic reagents to treat bone diseases caused by deregulated osteoclastogenesis, the signaling molecules and transcription factors under RANKL signaling axis for osteoclastogenesis have been extensively investigated.

Natural plant products are attracting increasing attention for their efficacy in the prevention and treatment of various metabolic diseases. The pumpkin plant (*Curcubita sp.*) is a ubiquitous edible vine. The species of pumpkins that are commonly available include *Cucurbita pepo* (most common), *Cucurbita maxima*, *Cucurbita stilbo*, and *Cucurbita Moschata* 9. There is evidence showing that *Curcubita sp.* have beneficial effects on a variety of diseases. For example, fermented *Cucurbita moschata* extract has been shown to have anti-obesity potential by suppressing body weight gain by regulating adipogenic transcriptional factors 10. Additionally, pumpkin seed extract has been reported to have estrogenic properties in a rat model as well as the potential to overcome symptoms caused by estrogen deficiency 11. *Cucurbita Moschata* Duch. has long been considered a health food in many countries such as Mexico, India, China, Brazil, and Korea 12. *Cucurbita Moschata* Duch. flesh and seeds are nutritionally rich due to the proteins and antioxidant vitamins such as carotenoids and tocopherols 13. Dehydrodiconiferyl alcohol (DHCA), a lignan originally isolated from *Cucurbita moschata*, is believed to be a phytoestrogen based on its structure and is also thought to prevent OVX-induced osteoporosis by inhibiting osteoclastogenesis 14. However, the inhibitory potential and molecular mechanisms of tendril of *Cucurbita Moschata* Duch. (TCMD) extract on RANKL-induced osteoclast differentiation have yet to be elucidated.

In this study, we investigated the inhibitory effect of TCMD extract on RANKL-induced osteoclast differentiation, provided molecular mechanisms explaining its inhibitory activity, and suggested possibilities for the use of TCMD as a traditional medicine against bone diseases.

## Materials and Methods

### Preparation of pumpkin tendril water extract

The dried tendril of pumpkin (*Cucurbita Moschata* Duch.) was purchased at the Jjanggu-oppa agricultural market (Jinju, Gyeongsangnam-do, Korea). The pumpkin tendril was identified by Prof. Kang-Mo Ku, Department of Horticulture Science, Chonnam National University. A voucher sample (no. JNUCM20180807) was deposited in the herbarium of the laboratory. The pumpkin tendrils (200 g) were washed well in purified water and then placed in the extractor (DW290, Daewoongbio, Seoul, Korea). After the addition of 1 L of distilled water, it was extracted at 90 °C for 4 h. The extracted samples were filtered through a glass fiber filter on a Buchner funnel and concentrated through vacuum evaporation (N1110 EYELA, Tokyo, Japan). The collected samples were stored at -20 °C for 48 h in a freezer (RB 603GMWP, LG, Seoul, Korea) and lyophilized (TFD8503 Ilshin Lab Co., Ltd, Seoul, Korea).

### Osteoclast differentiation and TRAP staining

Mouse bone marrow cells (BMCs) were isolated from the femurs of six-to-eight-week-old male C57BL/6 (KOATECH, Gyunggi-Do, Korea) 15. After lysing the red blood cells, the cells were incubated for three days in CO_2_ incubator in α-minimal essential medium (MEM) (Gibco) with 10% fetal bovine serum (Corning) containing 30 ng/ml M-CSF. The attached cells (BMDMs, bone marrow-derived macrophages) were used as osteoclast precursors by removing floating cells. For generation of osteoclasts, the BMDMs were seeded in 12-well plates with M-CSF plus RANKL for four days. Osteoclast formation was determined through tartrate-resistant acid phosphatase (TRAP) staining. For TRAP staining, the cells were fixed with a fixative solution and stained with a commercial kit (SIGMA) according to the manufacturer's instructions. After staining, TRAP-positive multinucleated cells (TRAP^+^MNCs) containing more than three nuclei were counted under a light microscope. All the animal experiments for primary bone marrow cells were performed according to the Institutional Animal Care and Use Committee (IACUC) of Chonnam National University (approval number: CNU IACUC-YB-2016-34).

### Cell viability assay

First, BMDMs were seeded in a 96-well plate with M-CSF (30 ng/ml) and treated with different concentrations of TCMD (0.125, 0.25, 0.5, and 1 mg/ml). After 24 h, the EZ-Cytox (Daeillab service, Seoul, Kr) solution (10 μl) was added to each well and the plate was incubated for 3 h in a CO_2_ incubator. An ELx808 microplate spectrophotometer reader (BioTek, Winooski, VT, USA) was used to measure the absorbance at 450 nm.

### Western blotting

For Western blotting, BMDMs were seeded in 12-well plate and incubated overnight. These cells were treated with TCMD in the presence or absence of RANKL. At the indicated time point after treatment, cells were lysed in a buffer containing 1% NP-40, 50 mM Tris (pH 7.4), 250 mM NaCl, 5 mM EDTA, 50 mM NaF, 1 Mm Na_3_VO_4_, 0.02% NaN_3,_ 2 mM dithiothreitol plus protease inhibitor (Roche, Mannheim, Germany) and phosphatase inhibitor (Sigma-Aldrich). Whole cell lysates were separated using SDS-PAGE and transferred onto nitrocellulose membranes The membranes were probed with primary antibodies against IκBα, phospho-form of p38, ERK, JNK, and p65 (Cell Signaling Technology, Danvers, MA, USA). A primary antibody for β-actin was obtained from Santa Cruz. The membranes were then washed with TBST and incubated with HRP-conjugated secondary antibodies (Santa Cruz, CA, USA) for 2 h at room temperature. The bands were visualized using Clarity Western ECL Substrate (Bio-Rad, Hercules, CA, USA) on a luminescent image analyzer (Amersham Imager 600, GE Healthcare, UK).

### Real-time quantitative polymerase chain reaction (qPCR)

Total RNA was isolated from osteoclasts using easy-Blue (iNtRON Biotechnology, Seongnam, Korea) and complementary DNA was synthesized from 1 μg RNA using an RT Premix reverse transcription system (AccuPower, Seoul, Korea). Then, qPCR was performed using cDNA as a template with Power SYBR Green (Applied Biosystems, Warrington, UK). β-actin was used for normalization. qPCR was performed for 40 cycles at 95 °C for 10 s, 60 °C for 60s using the Rotor-Gene Q real-time PCR system (Qiagen, Hilden, Germany).

The following primers were used: TRAP, 5'-CTGGAGTGCACGATGCCAGCGACA-3' and 5'-TCCGTGCTCGGCGATGGACCAGA-3'; DC-STAMP, 5'-CCAAGGAGTCGTCCATGATT-3' and 5'-GGCTGCTTTGATCGTTTCTC-3'; Cathepsin K, 5'-GGCCAACTCAAGAAGAAAAC-3' and 5'-GTGCTTGCTTCCCTTCTGG-3'; NFATc1, 5'-CTCGAAAGACAGTGGAGCAT-3' and 5'-CGGCTGCCTTCCGTCTCATAG-3'; and β-actin, 5'-AGGCCCAGAGCAAGAGAG-3' and 5'-TCAACATGATCTGGGTCATC-3'.

### Bone resorption assay

To observe osteoclast-mediated bone resorption *in vitro* 16, BMDMs were cultured for four days with M-CSF and RANKL in the presence or absence of TCMD on an Osteo assay surface 24-well plate (Corning Inc., NY). The medium was aspirated from the resorption assay plate and 20% sodium dodecyl sulfate (SDS) was added for 15 min to remove the cells. The plate was washed 3 times with distilled water and subsequent air drying. The areas absorbed on the discs were observed using a microscope (Eclipse Ni-U, Nikon, Japan).

### F-actin ring formation staining

In order to evaluate the formation of actin ring *in vitro* 16, BMDMs differentiated in four days with RANKL and TCMD on a cover glass. The cells were fixed with 4% paraformaldehyde and permeabilized with 0.2% Triton X-100/PBS for 10 mins, then stained with 0.1% Alexa Fluor 594-phalloidin (Invitrogen) and with DAPI (Invitrogen). The images were obtained using a fluorescence microscope (Korea lab tech, Korea).

### Statistical analysis

All statistical calculations were performed using GraphPad Prism version 5.01 (GraphPad, La Jolla, CA, USA). One-way ANOVA followed by Tukey's post-hoc test was used to assess the differences between specific groups. A result was considered statistically significant if the *P*-value was less than 0.05.

## Results

### Water extract of tendril of *C. Moschata* Duch*.* (TCMD) suppresses RANKL-induced osteoclast differentiation in BMDMs

**Figure 1:**
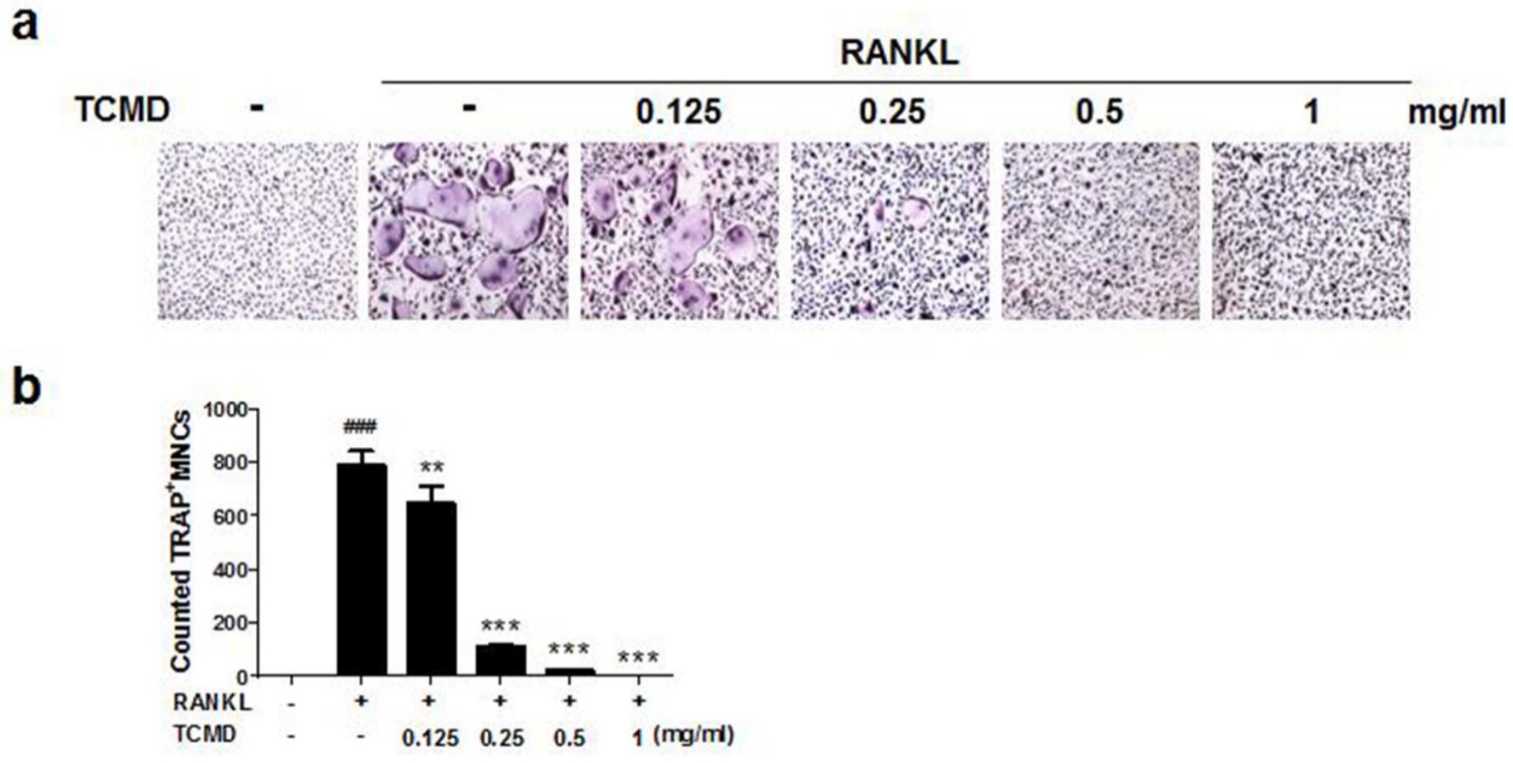
** TCMD suppresses RANKL-induced osteoclast differentiation in BMDMs. (A)** BMDMs were treated with M-CSF (30 ng/ml) and RANKL (100 ng/ml) for four days at the indicated concentration of TCMD. Multinucleated cells were visualized by TRAP staining. **(B)** TRAP-positive multinucleated cells were counted to determine osteoclast numbers. The results are expressed as mean ± SD. ^###^*p* < 0.001 vs. control , **p < 0.01, ***p < 0.001 vs. RANKL group.

**Figure 2:**
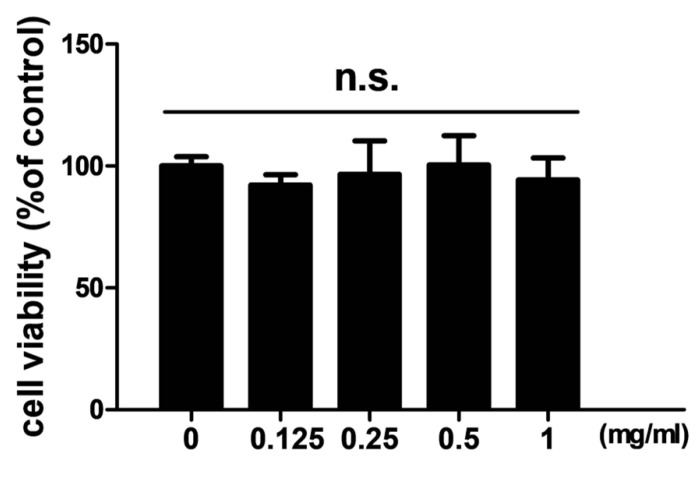
** Effect of TCMD extract on BMDMs. BMDMs** were cultured on a 96-well plate for 24 h in the presence of M-CSF and treated with the indicated concentration of TCMD. Cell viability was determined as described in the Materials and Methods. The results are expressed as mean ± SD.

In order to examine the effects of TCMD on RANKL-induced osteoclast differentiation, BMDMs were treated with various concentrations of the water extract of TCMD in the presence of M-CSF and RANKL. Treatment with RANKL effectively differentiated the BMDMs of the control group into TRAP-positive mature multinucleated osteoclasts, as shown by TRAP staining. By contrast, the water extract of TCMD decreased the formation of osteoclast from BMDMs in a dose-dependent manner (Fig. 1A and B). To confirm whether the inhibitory effect of TCMD can be attributed to the toxicity of this extract, we examined cell viability assays in the culture after 24h. The results show that, compared to the control treatment, TCMD had no cytotoxic effect on BMDMs at concentrations below 1 mg/ml (Fig. 2).

### Water extract of tendril of *C. Moschata* Duch*.* (TCMD) inhibits the formation of F-actin ring and bone resorption activity of mature osteoclasts

**Figure 3:**
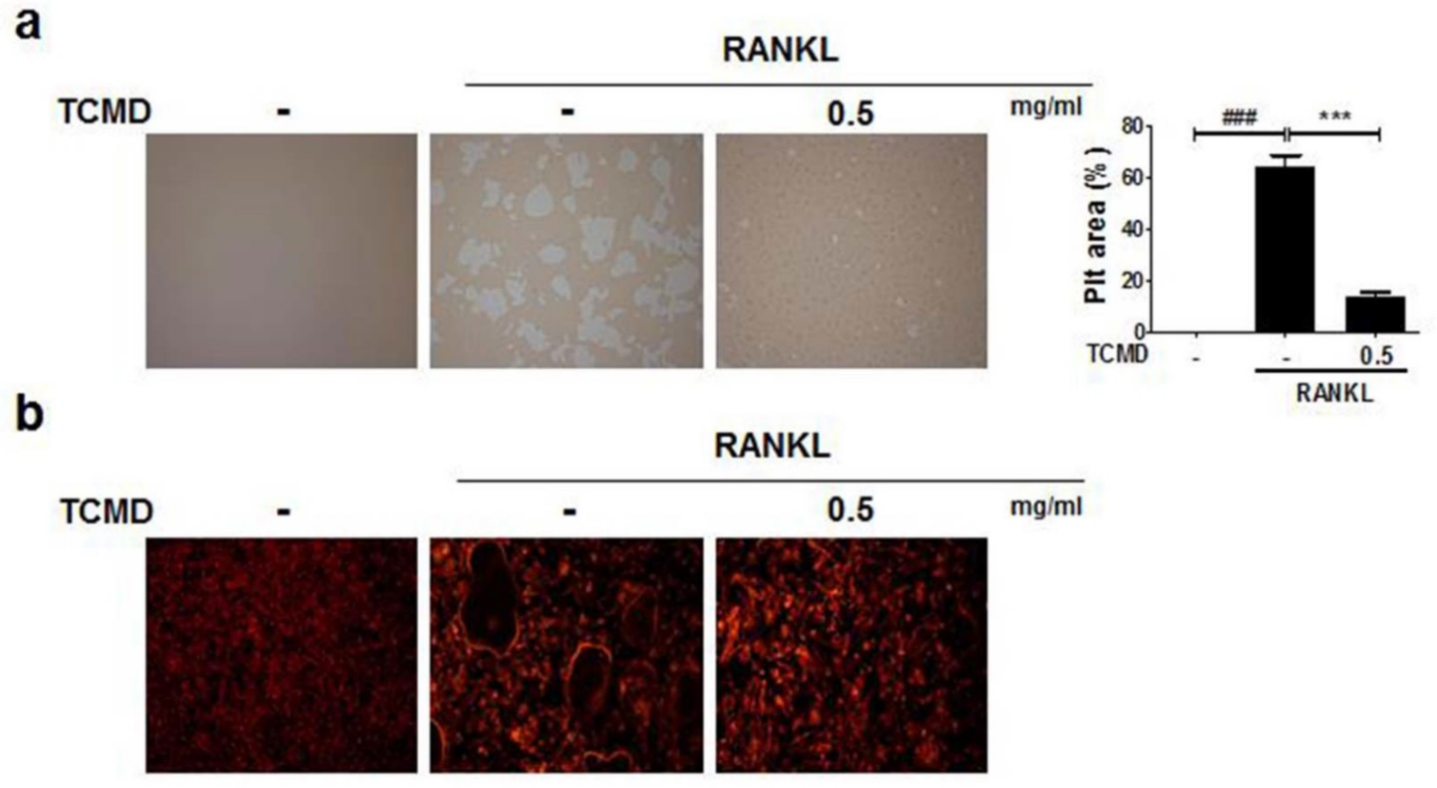
** TCMD inhibits F-actin ring formation and osteoclastic bone resorption. (A)** BMDMs were seeded on Osteo Assay Surface Plates and pretreated with TCMD (0.5 mg/ml) for 1 h, then the cells were cultured with M-CSF (30 ng/ml) and RANKL (100 ng/ml) for an additional four days. **(B)** BMDMs were incubated with M-CSF (30 ng/ml) and RANKL (100 ng/ml) in the presence or absence of TCMD (0.5 mg/ml) for four days. The cells were fixed and stained for F-actin ring with Alexa-Fluor 594 phalloidin and imaged on a confocal system. Pit area was obtained based on the percentage of RANKL-induced destructive area obtained using image J program. Data are presented as mean ± SD. ^###^*p* < 0.001 vs. control; ***p < 0.001 vs. RANKL group.

As TCMD suppressed osteoclast differentiation, we examined whether the extract of TCMD has the potential to inhibit the function of osteoclasts derived from BMDMs. In order to examine the effect of TCMD on osteoclastic bone resorption, BMDMs were cultured on an Osteo assay surface plate with various concentrations of TCMD in the presence or absence of RANKL for three days, and the resorbed area was observed under a light microscope. Then, the number and area of resorption pits remarkably decreased in the TCMD-treated group (Fig. 3A). The F-actin ring in osteoclasts is essential for bone resorption. Therefore, we visualized the actin cytoskeleton of differentiated BMDMs through rhodamine-phalloidin staining. Fluorescence microscopy revealed that the F-actin ring structure was abolished by TCMD treatment in the RANKL-treated cells (Fig. 3B).

### Water extract of tendril of *C. Moschata Duch.* (TCMD) down-regulates RANKL-induced NF-κB and MAPKs activation in vitro

**Figure 4:**
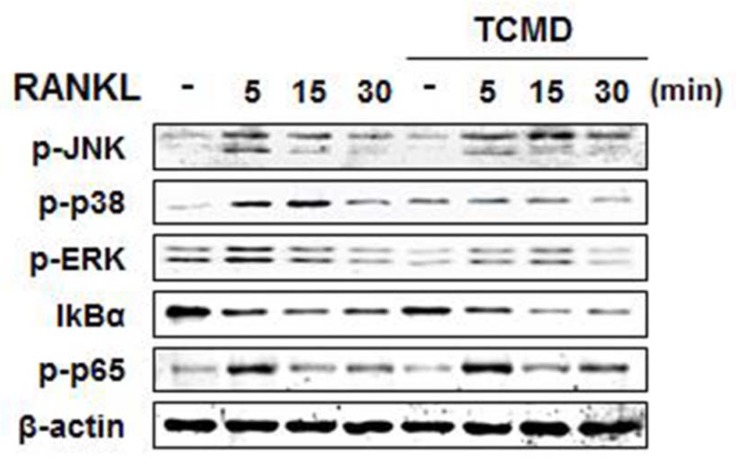
TCMD blocks RANKL-induced activation of p38 and ERK. BMDMs were pretreated with or without TCMD (0.5 mg/ml) for 1 h and then treated with RANKL (100 ng/ml) for the indicated times. Whole cell lysates were analyzed by Western blot with the indicated antibodies. The uncropped Western blots are presented in Supplementary Figure S1.

RANKL binding to its receptor, RANK, activates MAP kinases (ERK, JNK, and p38) signaling pathways, transferring RANKL signaling to the related transcriptional factors for osteoclast differentiation 17. In order to investigate the molecular mechanism by which TCMD suppressed RANKL-induced osteoclastogenesis, we examined the inhibitory effects of TCMD on RANKL-induced early signaling events, such as NF-κB and MAPKs signaling pathways. First, BMDMs were pretreated with TCMD for 2 h and subsequently simulated with RANKL in the presence of TCMD for the indicated time points. The stimulation of BMDMs with RANKL increased the phosphorylation levels of ERK, p38, JNK, and p65, as well as the degradation of IκBα. However, pretreatment with TCMD significantly decreased the phosphorylation levels of p38 and ERK, not JNK (Fig. 4). By contrast, TCMD did not block the RANKL-induced degradation of IκBα protein, suggesting that it could not inhibit RANKL-induced NF-κB activation (Fig. 4).

### Water extract of tendril of *C. Moschata* Duch. (TCMD) suppresses RANKL-induced gene expression of osteoclastogenesis-related factors

**Figure 5:**
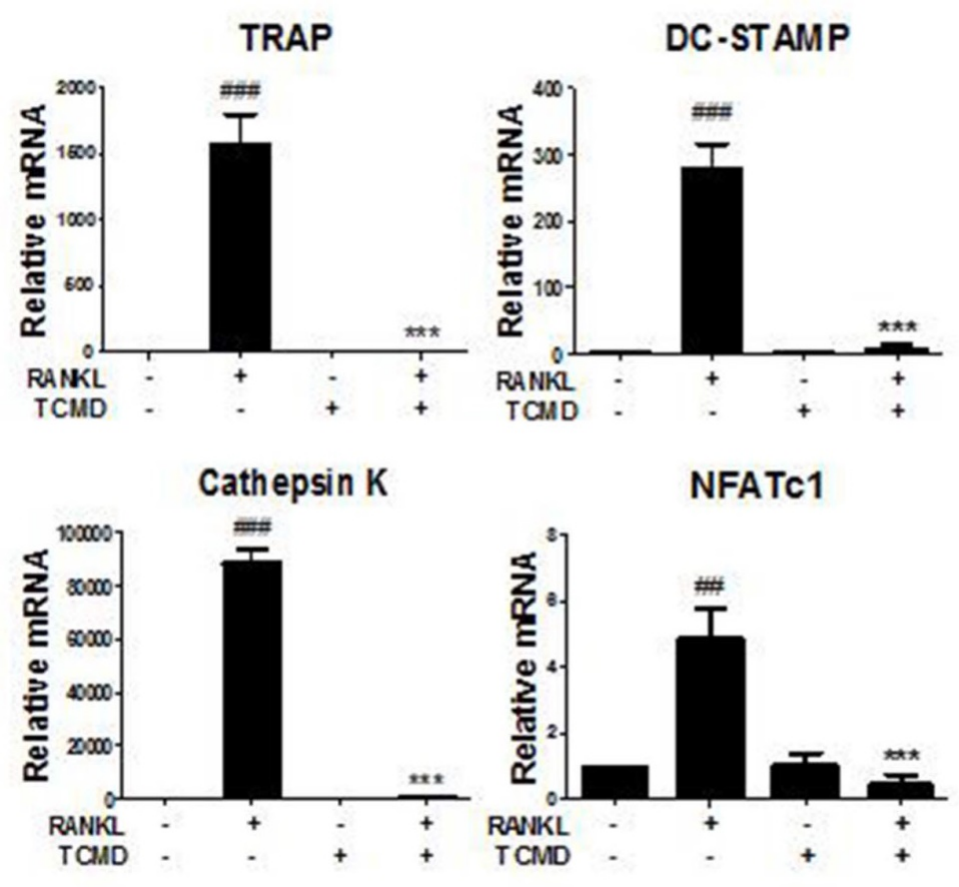
TCMD regulates RANKL-mediated osteoclast-specific gene expression. BMDMs were pretreated with or without TCMD (0.5 mg/ml) for 1 h and then treated with RANKL (100 ng/ml) for four days. Osteoclast-specific gene expression was analyzed using real-time PCR and results were normalized to the expression of β-actin. ^##^*p* < 0.01, ^###^*p* < 0.001 vs. control, ***p < 0.001 vs. RANKL-treated group.

We further investigated whether TCMD influences the RANKL-induced gene expression such as TRAP, osteoclast-associated receptor (OSCAR), Cathepsin K, dendritic cell-specific trans-membrane protein (DC STAMP), and NFATc1. The results of real-time PCR analysis showed that the expressions of all of the tested genes were upregulated by RANKL, but that they were mostly abolished by TCMD treatment (Fig. 5).

## Discussion

Excessive osteoclast activity causes many diseases, and to date, anti-osteoporosis therapy has targeted this cell 18. Therefore, inhibiting the formation of bone-resorbing osteoclasts through the suppression of RANKL signaling or its downstream pathways should be a rational target for the treatment of osteopenic diseases like osteoporosis. The pumpkin plant (*Curcubita sp.*) has long been used in traditional Asian medicine. This experiment is a new report investigating the anti-osteoclastogenesis effects of pumpkin tendril extract.

In the present study, we demonstrated for the first time that the water extract of TCMD, tendril of *Cucurbita Moschata* Duch., exerted an inhibitory effect on RANKL-induced osteoclastogenesis. BMDMs are osteoclast precursor cells that differentiate into osteoclasts in response to RANKL, which is expressed in osteoblasts, bone and lymphoid tissues, and is also a critical factor in osteoclastogenesis 19. RANK/RANKL interaction forms a multiple intracellular cytokine system that regulates osteoclast differentiation 20. In our study, TRAP activities were measured in RANKL-induced BMDMs in order to evaluate the effects of TCMD on osteoclast differentiation. RANKL induced the development of multinucleated osteoclasts from precursors, and TCMD treatments exerted preventive effects on the formation of the TRAP-positive osteoclast without any cytotoxicity, as well as actin ring formation and bone resorption. Our previous study reported that rutin was abundant in the extract of TCMD, which had an anti-inflammatory effect 21. Additionally, a recent study indicated that rutin had preventive effects against RANKL-induced osteoclast differentiation by inhibiting ROS production 22. This is likely because TCMD contains rutin, resulting in a stronger inhibitory effect on osteoclast differentiation.

The connection between RANK and RANKL induces the recruitment and activation of TRAF6, which can regulate NF-κB and MAPKs signaling pathways 23. NF-κB regulates osteoclast formation and activetion in bone resorption during RANKL-induced osteoclast signaling 24. RANKL-induced NF-κB activation leads to the degradation of IκBα, followed by nuclear translocation of the NF-κB subunit p65 and binding with DNA target sites 25. In addition, it has been well established that the inhibition of MAPKs can suppress RANKL-induced osteoclast formation. RANKL-induced p38 and JNK phosphorylation is necessary for osteoclast differentiation signaling in BMDM 26,27. However, ERK activation is more responsible for osteoclast survival than osteoclast differentiation 28. In the present study, we found that TCMD inhibited osteoclast differentiation by suppressing RANKL-mediated p38 and ERK phosphorylation and observed no evidence that TCMD could inhibit RANKL-induced JNK and p65 phosphorylation during osteoclastogenesis. However, the detailed mechanism by which TCMD suppresses RANKL-induced activation of MAPKs remains to be elucidated.

Activated MAPKs stimulate a variety of downstream targets including c-Fos and NFATc1, essential for the regulation of osteoclastogenesis in response to M-CSF and RANKL 29. In particular, the amplification of NFATc1, a master regulator of osteoclast differentiation, regulates osteoclast-related genes such as cathepsin K, TRAP 30, 31. DC-STAMP is an important regulator of cell fusion, and the fusion of mononuclear cells in bone resorption triggers the formation of multinucleated osteoclasts 32. In this study, the expressions of all genes related to osteoclastogenesis, including TRAP, DC-STAMP, cathepsin K, and NFATc1, were significantly down-regulated by TCMD treatment. Consequently, TCMD may exert anti-osteoporotic effects by inhibiting the expression of the genes involved in osteoclastogenesis.

## Conclusions

In summary, this is the first study to report that TCMD efficiently suppresses RANKL-induced osteoclast differentiation and bone-resorbing activity in vitro. Regarding molecular mechanisms, we showed that TCMD inhibits the RANKL-induced activation of p38 and ERK and down-regulates the expression of osteoclast-specific genes. Our results suggest that TCMD may be an effective traditional therapeutic medicine for the treatment of bone diseases. Further study is recommended to investigate the potential clinical application of TCMD in postmenopausal women with osteoporosis.

## Supplementary Material

Supplementary figure S1.
