# Supplementary figures and images for: Water extract of tendril of Cucurbita Moschata Duch. suppresses RANKL-induced osteoclastogenesis by down-regulating p38 and ERK signaling
Source: Int J Med Sci. 2020 Feb 24;17(5):632–9. doi: 10.7150/ijms.39622 (PMC7085206; doi:10.7150/ijms.39622)

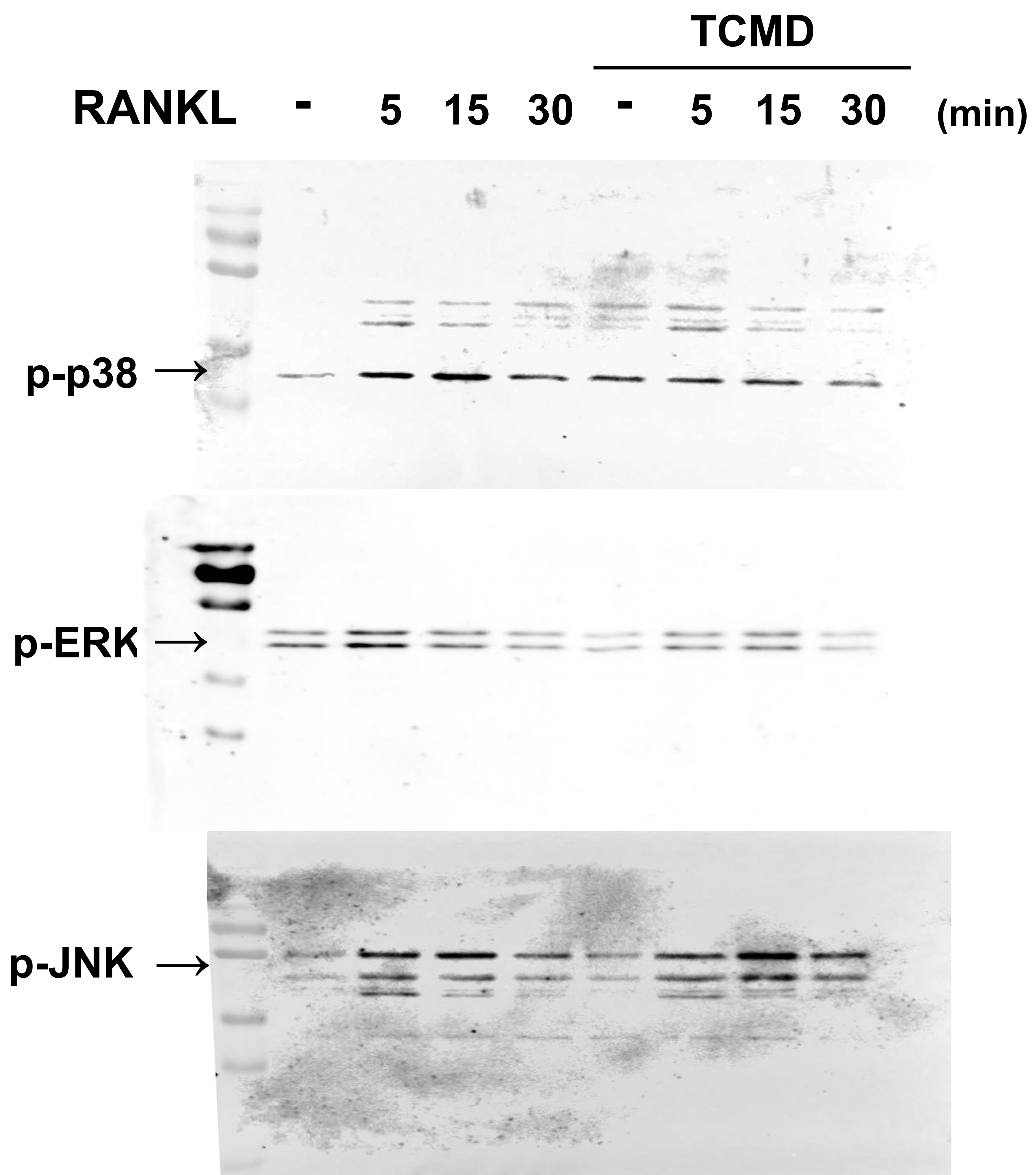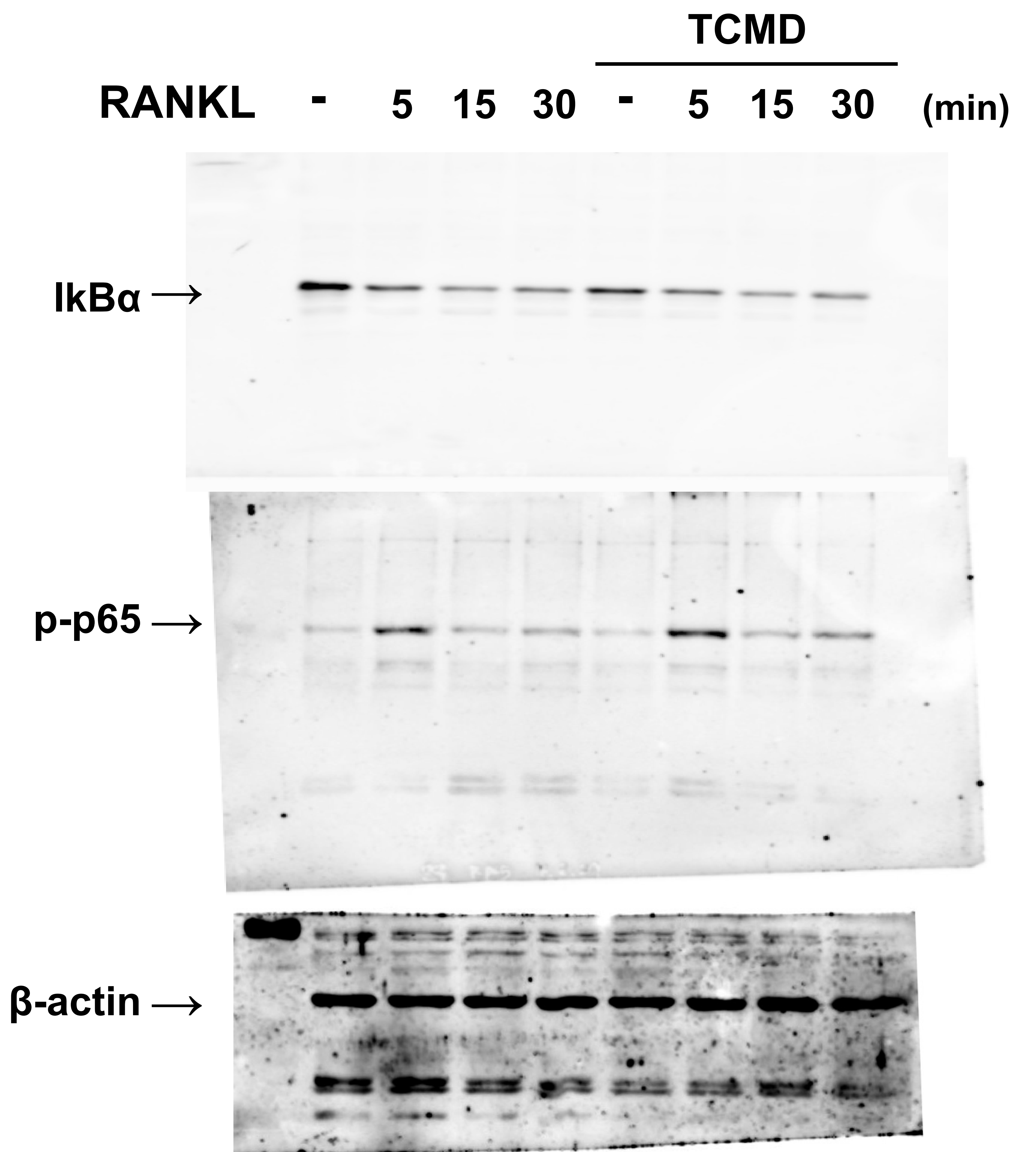

Supplement: Supplementary file 1 — Supplementary figure S1. [file ijmsv17p0632s1.pdf]
